# Extreme Differences in Forest Degradation in Borneo: Comparing Practices in Sarawak, Sabah, and Brunei

**DOI:** 10.1371/journal.pone.0069679

**Published:** 2013-07-17

**Authors:** Jane E. Bryan, Philip L. Shearman, Gregory P. Asner, David E. Knapp, Geraldine Aoro, Barbara Lokes

**Affiliations:** 1 School of Geography and Environmental Studies, University of Tasmania, Hobart, Tasmania, Australia; 2 UPNG Remote Sensing Centre, University of Papua New Guinea, Waigani, National Capital District, Papua New Guinea; 3 Research School of Biology, Australian National University, Canberra, Australian Capital Territory, Australia; 4 Department of Global Ecology, Carnegie Institution for Science, 260 Panama Street, Stanford, California, United States of America; Centre National de la Recherche Scientifique, France

## Abstract

The Malaysian states of Sabah and Sarawak are global hotspots of forest loss and degradation due to timber and oil palm industries; however, the rates and patterns of change have remained poorly measured by conventional field or satellite approaches. Using 30 m resolution optical imagery acquired since 1990, forest cover and logging roads were mapped throughout Malaysian Borneo and Brunei using the Carnegie Landsat Analysis System. We uncovered ∼364,000 km of roads constructed through the forests of this region. We estimated that in 2009 there were at most 45,400 km^2^ of intact forest ecosystems in Malaysian Borneo and Brunei. Critically, we found that nearly 80% of the land surface of Sabah and Sarawak was impacted by previously undocumented, high-impact logging or clearing operations from 1990 to 2009. This contrasted strongly with neighbouring Brunei, where 54% of the land area remained covered by unlogged forest. Overall, only 8% and 3% of land area in Sabah and Sarawak, respectively, was covered by intact forests under designated protected areas. Our assessment shows that very few forest ecosystems remain intact in Sabah or Sarawak, but that Brunei, by largely excluding industrial logging from its borders, has been comparatively successful in protecting its forests.

## Introduction

Over the last several decades, tropical forests have been cleared and degraded at an accelerating rate, with losses contributing to what is likely to be the sixth global mass extinction in Earth’s history [1]. Deforestation refers to the replacement of forests with different land cover types such as crops or grassland, and forest degradation refers to the substantial reduction of biomass, usually by the removal of big trees, whilst retaining sufficient tree cover to still be classified as ‘forest’. Logging and fire are the major causes of forest degradation in the tropics. Between 2000 and 2005, roughly 27 million hectares of forest in the tropics were cleared [2], largely for timber or agricultural plantations or crops, and over much the same time period, approximately 398 million hectares were allocated to the industrial logging industry [3].

The loss and degradation of tropical forests is of great concern because these systems are among the most biodiverse places remaining on Earth - they provide habitat for many species, contain a rich array of plant and animal life not found elsewhere, and play a major role in regulating local as well as global climate and weather patterns [4]–[7]. Large rainforest trees are often long lived, with ages commonly exceeding many hundreds of years [8]–[13]. These big trees are important for ecosystem health, providing a source of seeds and fruits for species propagation, as well as habitat for a wide range of other organisms [12]–[14]. Degradation of primary forest ecosystems, especially by logging, results not only in the disproportionate loss of large trees and the ecosystem functions they provide, but also causes substantial collateral damage to residual vegetation, carbon emissions, damage to soils and waterways, with repeated harvests resulting in progressive degradation [14]–[19]. Intact forests, or forests that have not been degraded, are central to sustaining biodiversity [7].

The Malaysian states of Sabah and Sarawak are global hotspots of tropical deforestation [2]. These two states in the north of the island of Borneo are also centers of the tropical oil palm and industrial logging industries, with Sarawak in particular being the place of origin for many Malaysian logging companies that now operate in Papua New Guinea, The Solomon Islands, tropical Africa and Guyana, amongst other places. In many countries these companies are responsible for unsustainable harvesting and short-term profit maximization [20]. Unsustainable and damaging logging practices, often followed by the conversion of logged forest to oil palm and timber plantations, are particular problems in Sabah and Sarawak [2], [15]–[17], [21].

In contrast to the situation in Sabah and Sarawak, the neighbouring petroleum-rich nation of Brunei has charted a different path, shunning wide-scale intensive logging and oil palm plantations in favor of preserving forest ecosystems. Given the known differences in agro-timber industries among these jurisdictions, it is timely to examine the condition of forests in these regions, and the outcome of the alternative forest-protecting pathway taken by Brunei.

Recent wall-to-wall assessments of forest area and land cover of the northern part of Borneo have been conducted using medium resolution optical satellite imagery (250–500 m resolution), and in some instances combined with radar [2], [21]–[22]. However, logging cannot be reliably detected using these data making it difficult to distinguish intact from degraded forest where forest cover remains. Thus whilst the area of oil palm and the overall area of forest (woody vegetation with some form of closed canopy) have been measured in Malaysian Borneo and Brunei [21]–[22], the condition of the forests remaining in these regions has not been assessed.

In recent years, a number of different methods have been developed that allow forest cover and deforestation to be assessed, but very few approaches can also detect and map forest degradation. One of the few methods able to do so is the Carnegie Landsat Analysis System (CLAS) and its successor CLASlite [23], which has been used to map deforestation and forest degradation throughout the Amazon basin, Madagascar, and elsewhere [3], [23]. Another of the methods involves manual or semi-automated classification of forest cover from optical remotely-sensed imagery, with proximity to roads used as a measure of forest condition [24]–[25].

In our analysis we combined the CLASlite and semi-manual road mapping techniques to produce the first long-term, spatially-detailed analysis of intact and degraded forests throughout Malaysian Borneo and Brunei. Using CLASlite and the mapping of individual roads, we quantified both the area of forest and the condition of the forest, whether relatively intact forest ecosystems, degraded forest cover, or forests that have been severely degraded from either repeated logging over 20 years or from repeated clearances during the same period.

## Methods

### Study Area

The Malaysian states of Sabah and Sarawak and the independent nation of Brunei are the focus of this study, with emphasis on natural (unplanted) forests. Natural forests in this region are composed primarily of mixed Dipterocarpaceae and peat swamp species. The legal commercial logging of these forests in Sabah and Sarawak is termed ‘selective harvesting’ or ‘selective logging’. Under this logging regime, the majority of commercial *Dipterocarps* (>60 cm diameter at breast height) are felled and harvested in the first cycle (>45 cm in Sarawak for non-*Dipterocarp* species), generally yielding 50–150 m^3^ha^−1^ of timber in the first harvest, with the aim of securing sufficient regeneration of commercial trees to allow a second harvest 25–30 years later [15], [17], [26]. Substantial damage to soil, waterways and forest structure and residual trees is caused by this form of logging, with progressive degradation of biomass over repeated harvest cycles [15], [17], [27]. Bulldozers impact approximately 30–40% of the logged area and damage is caused to 40–70% of residual trees [15]. For these reasons initial timber yields cannot be maintained over multiple harvest cycles, with 25–30 years between harvests too short a period to allow regeneration of timber stocks [15], [17], [27].

We used the CLASlite forest monitoring system [23] to measure land cover with closed canopy tree or palm cover using orthorectified Landsat imagery from the year 2009. We then spatially separated forest ecosystems from forest that had been logged once, repeatedly logged forest, mangroves, tree plantations, palm plantations or regrowth that had been cleared at least once since 1990. This was done using digital maps of roads constructed in the period from 1990 to 2009 that we produced by digitizing roads apparent in Landsat (described below), and from manually digitizing areas of clearance discernible in imagery over the same period.

### Map of Tree/palm Cover 2009

We used the CLASlite system to produce a 2009 base map depicting the extent of land area with a closed tree or palm canopy. This map was generated from Landsat images covering Sabah, Sarawak and Brunei. Imagery from a single year could not be used to generate the map due to persistent cloud cover. Seventy nine percent of images used came from the period 2007–2009, with 47% recorded in 2009. Consequently hereafter we refer to the coverage as ‘2009’. The remaining 21% of images were recorded in 2001, 2004 or 2005. Each satellite image was orthorectified using 10–15 ground control points and a Digital Elevation Model (DEM) at 90-m spatial resolution obtained from the Shuttle Radar Topography Mission (SRTM, [28]). The average root mean square error for the orthorectified imagery was 25–30 m. The date and image path and row used to produce the ‘2009’ tree/palm cover map is shown in Table S1.

CLASlite uses raw satellite imagery to produce forest cover and forest cover change images. In this analysis we used only the forest cover component of CLASlite. CLASlite performs an automated radiometric calibration and atmospheric correction on the input imagery, masks out cloud, water and shadow, and then decomposes remaining image pixels into fractional cover using an Automated Monte Carlo Unmixing algorithm which compares the spectral signature of an image pixel on 4–7 image bands with spectral signature libraries derived from extensive field surveys [23].

Each image pixel is decomposed into fractional photosynthetic (live) vegetation cover (PV), non-photosynthetic vegetation (dead) cover (NPV), and bare substrate (BS) [23]. Measurement of forest disturbance using CLASlite and its precursors have been validated in Brazil and Peru [18], [29], as well as Indonesian Borneo and Hawaii [23]. A threshold value of 80% photosynthetic vegetation has previously been used to delineate ‘forest’ from ‘non-forest’ across a range of different tropical regions [23]. In Sabah and Sarawak however, preliminary comparisons with the input imagery revealed that the 80% PV threshold did not distinguish between mangrove forests, oil palm, timber plantation and natural forest. We included an additional threshold of no more than 15% bare substrate which excluded younger oil palm and timber plantations from the ‘forest’ category, but did not separate mangrove forests, older timber and mature oil palm plantations. We therefore refer to the initial CLASlite map of land cover with greater than 80% PV and less 15% bare substrate as a map of ‘2009 tree/palm cover’ in which there are two classes – ‘tree/palm cover’ (>80% PV and <15% BS) and non-forest (all other values).

To separate timber, oil palm plantations, young regrowth after clearfell and mangroves from intact forest, we manually digitised mangroves, plantations and cleared areas from the imagery (listed in Table S1), and from imagery recorded from earlier dates covering the same areas. We aimed to acquire imagery covering the 1990 s and the 2000 s from which to map cleared areas and plantations in addition to the ‘2009’ imagery used to generate the base map (Table S1). However, perpetual cloud cover meant that annual imagery over a consistent time period was not possible across all areas. With the exception of one image (path:row 118∶55) all areas were covered by at least two images. The images and date range covering each path and row from which cleared areas and plantations were digitized are shown in Table S2. We then manually digitized mangroves, the areas of clearance and the areas covered by plantations, and areas of intensive drainage and conversion of peat swamp forests. Logging of peat swamp forests initially appears as a distinctive criss-cross ‘railway’ pattern of canals or timber rails that are constructed to facilitate the removal of timber from these systems. Examples of the peat swamp drainage and logging are contained in the Supporting Information S1. Any area classified as ‘tree/palm cover’ in the 2009 CLAS map that was subsequently identified as plantation or intensive peat swamp conversion during the manual digitization process was reclassified as ‘plantation/regrowth’. Any area classified as ‘tree/palm cover’ in the 2009 CLASlite map that had previously been cleared but had not been replaced with plantation was also reclassified as ‘plantation/regrowth’. Any area classified ‘tree/palm cover’ identified as mangrove forests was reclassified as ‘mangroves’. All other areas classified as ‘tree/palm cover’ in the map were reclassified as ‘forest’. Major regions covered by cloud in the resultant classification were manually filled using images from after 2005 listed in Table S2.

**Figure 1:**
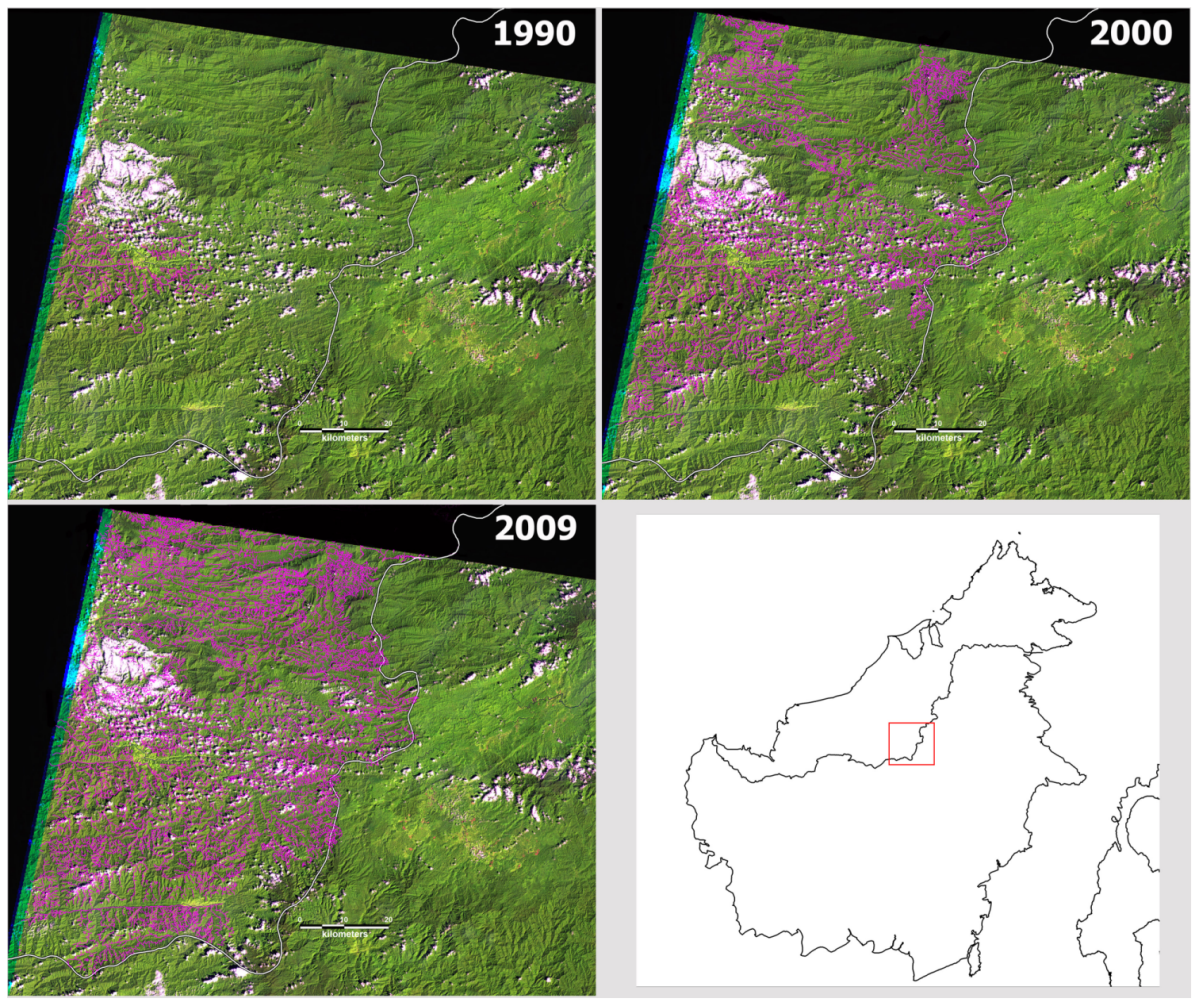
Landsat images showing successive roads (in pink) built between 1990 and 2009 in a forested region of the ‘Heart of Borneo’, Sarawak.

Roads were manually digitized from all images in Table S1 and Table S2, and each road was classified according to the year in which it first appeared in the imagery. In mountainous regions where recent logging is apparent in the imagery, we measured skid trails and canopy gaps extending approximately 350 metres from the feeder roads. In flatter terrain, we measured logging activity usually extending much further - up to 1 km or more. We therefore took a conservative approach and defined forest within 350 m of a road as having been logged. Illustrations of the conservative nature of this 350 m buffer are illustrated in the Supporting Information S1. We then combined roads built in the same year into a single layer and buffered each single road layer by 350 m. In many locations where repeated harvest cycles of ‘selective logging’ has occurred, multiple roads have been built through the same area of forest at different times between 1990 and 2009 (see Figure 1). We combined each road buffer from individual years into one multi-year road buffer layer to create a ‘logging intensity’ map. Locations through which a single road was built between 1990 and 2009 were given a value of 1, while locations where multiple roads had been built over the same time period were given a value greater than 1.

We then combined the ‘logging intensity’ map with the ‘forest’ map derived from 2009 CLASlite tree/cover map and manually digitised plantations and regrowth to create a map of forest condition in 2009. All areas classified as ‘forest’ in 2009 that were within 350 m of a road were deemed to have been logged. All areas classified as forest that were beyond 350 m of a road were deemed to be ‘intact forest’. Forests within 350 m of a road with a logging intensity value of 1 are considered to have been logged once and are referred to as ‘degraded’ forests. Forests with a logging intensity value greater than 1 having been logged repeatedly since 1990, are considered to be ‘severely degraded’ forests. This approach is conservative with respect to logging intensity, as many areas where only one road has been built would have been harvested repeatedly.

In addition to assessing forest area and condition across Sabah, Sarawak and Brunei, we also assessed these parameters within the gazetted protected areas. Protected areas in Sarawak were defined according to the Forest Department of Sarawak [30] as forests within ‘Totally Protected Areas’ (TPAs), that is, National Parks, National Reserves and Wildlife Sanctuaries. The Sarawak Forestry Corporation [31] lists 18 National Parks, 5 Nature Reserves and 4 Wildlife Sanctuaries within Sarawak. Boundaries for all but three of these TPAs were obtained from the World Database on Protected Areas [32]. Boundaries for the Kuching Wetland National Park (6610 ha), Wind Cave Nature Reserve (6 ha), and Sama Jaya Nature Reserve (38 ha) were unavailable, but made up only 1.2% of the area of all TPAs [31], and hence had little impact on the final statistics for protected areas in Sarawak. Protected areas in Sabah were defined according to the Sabah Forestry Department [30] as Protected, Domestic, Amenity, Wildlife and Virgin Jungle Reserves. In addition, National Parks, current Bird Sanctuaries, Mangrove Forest Reserves and State Parks were also included as ‘protected areas’. Boundaries for each of these protected areas were obtained from the World Database on Protected Areas (Figure S1, [32]).

## Results

**Figure 2:**
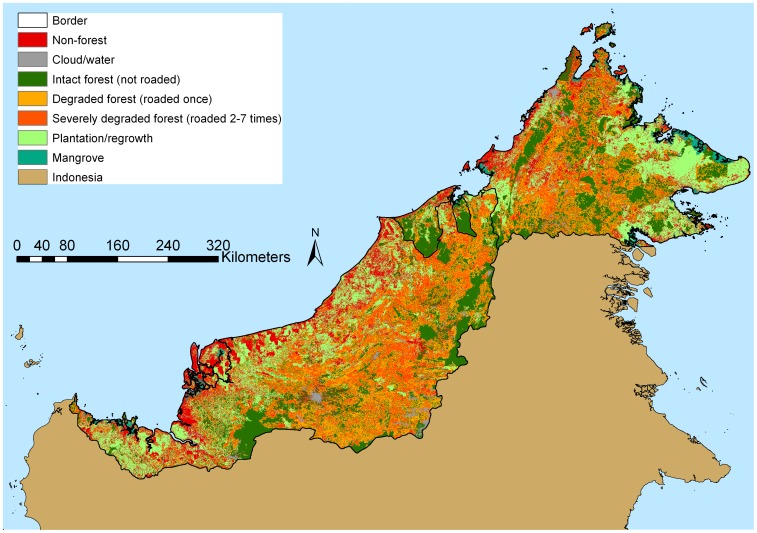
Forest cover and condition in Malaysian Borneo and Brunei in 2009. Intact forest, degraded forest, mangroves, plantations and regrowth are shown.

A total of 364,489 km of logging roads were built in Malaysian Borneo and Brunei between 1990 and 2009. If these roads were placed end-to-end would circle Earth nine times. The final forest condition map showing the extent of intact and degraded forest, mangroves, plantations and regrowth is shown in Figure 2. The 2009 ‘tree/palm cover’ for Sabah, Sarawak and Brunei created from CLASlite fractional cover is shown in shown in Figure S2 in the supporting information, and the ‘forest’ map, irrespective of condition, with mangroves, plantations and regrowth on previously cleared land (since 1990 and/or the 2000 s) is shown in Figure S3.

The area of intact forest, logged forest, mangroves and plantations/regrowth is shown in Table 1. In total, just 22% of the land surface of Sabah and Sarawak remained as intact forest unlogged between 1990 and 2009. This estimate contrasts strongly with Brunei, where 54% of the land area was covered by intact, unlogged forests. Of the severely degraded forests (logged 2–7 times) (Table 1), in Sarawak 77% had been logged twice, 19% three times, and the remaining 3% four or more times. In Sabah 86% of severely degraded forests had been logged twice, 12% three times, and the remaining 1% four or more times; in Brunei 83% of the severely degraded forests were logged twice, 16% 3 times, and the remaining 1.6% four or more times. An example of successive road-building over time is shown in Figure 1.

**Table 1:** Land cover in 2009 in Sabah, Sarawak and Brunei mapped using CLASlite.

	Sabah		Sarawak		Sabah and Sarawak		Brunei		TOTAL	
Land cover type	Area(km^2^)	% LandArea	Area(km^2^)	% LandArea	Area(km^2^)	% LandArea	Area(km^2^)	% LandArea	Area(km^2^)	% LandArea
Not forest	10,884	15	23,301	19	34,185	17	786	13	34,971	17
Cloud/water	1,699	2	3,334	3	5,034	3	112	2	5,146	3
Intact Forest^1^	18,394	25	23,836	20	42,230	22	3162	54	45,391	23
Degraded forest^2^	15,734	21	26,852	22	42,587	22	619	11	43,206	21
Severely Degraded forest^3^	7,277	10	17,783	15	25,060	13	238	4	25,298	13
Plantation/regrowth	17,093	23	26,057	21	43,150	22	804	14	43,955	22
Mangrove	2,622	4	855	1	3,478	2	101	2	3,579	2
**TOTAL**	**73,705**		**122,019**		**195,724**		**5822**		**201,546**	

1 Not roaded;

2 Roaded once;

3 Roaded 2–7 times.

In Sabah, intact forests under protected areas covered 8% of the land surface, or 9% including mangroves, and these protected areas contained 56% cover of intact forest (Table 2). In Sarawak, only 3% of the land area was intact forest under protected areas, and these protected areas contained 72% cover of intact forest (Table 2). Our definition of ‘intactness’ does not preclude these forests from being logged prior to perhaps 1970, giving them time to have overgrown roads – however this will have occurred in only a small percentage of the overall intact area because most of the forests that were accessed in the post-war decades were subsequently cleared [33].

**Table 2:** Land cover in 2009 under protected areas (PA) in Sabah and Sarawak.

	Sabah						Sarawak						Sabah and Sarawak					
	Inside PA			Outside PA			Inside PA			Outside PA			Inside PA			Outside PA		
Land cover type	Area(km^2^)	% LandArea	% ofPA	Area(km^2^)	% LandArea	% ofNon-PA	Area(km^2^)	% LandArea	% ofPA	Area(km^2^)	% LandArea	% ofNon-PA	Area(km^2^)	% LandArea	% ofPA	Area(km^2^)	% LandArea	% ofNon-PA
Not forest	741	1.0	7.1	10,144	14	16	308	0.3	6	22,993	19	20	1,049	0.5	6.9	33,136	16.9	18.4
Cloud/water	268	0.4	2.6	1,431	2	2	39	0.0	1	3,295	3	3	307	0.2	2.0	4,727	2.4	2.6
Intact Forest^1^	5782	7.8	55.5	12,612	17	20	3536	2.9	72	20,300	17	17	9,319	4.8	60.9	32,911	16.8	18.2
Degraded forest^2^	1633	2.2	15.7	14,101	19	22	310	0.3	6	26,542	22	23	1,944	1.0	12.7	40,643	20.8	22.5
Severely degraded forest^3^	395	0.5	3.8	6,882	9	11	140	0.1	3	17,643	14	15	535	0.3	3.5	24,525	12.5	13.6
Plantation/regrowth	764	1.0	7.3	16,329	22	26	517	0.4	11	25,540	21	22	1,281	0.7	8.4	41,869	21.4	23.2
Mangrove	832	1.1	8.0	1,790	2	3	44	0.0	1	811	1	1	876	0.4	5.7	2,602	1.3	1.4
**TOTAL**	**10,415**	**14.1**		**63,289**	**86**		**4,895**	**4.0**		**117,124**	**96.0**		**15,311**	**7.8**		**180,413**	**92.2**	

1 Not roaded;

2 Roaded once;

3 Roaded 2–7 times. PA = Protected Area.

## Discussion

Our high-resolution analysis measured an aspect of forests in Malaysian Borneo and Brunei that has not previously been assessed: forest condition. We conservatively estimate that in 2009 there were 45,390 km^2^ of intact forests in Malaysian Borneo and Brunei, covering just 22% of land area in Sabah and Sarawak, and 54% of land area in Brunei. Of the total remaining forest cover in Malaysian Borneo (109,877 km^2^ in 2009), only 38% remains relatively intact. The remainder is either degraded (39%) or severely degraded (23%). This demonstrates that large forest areas are being logged repeatedly with rotations well under the 60 years that is prescribed in most Malaysian forestry plans. In contrast in Brunei, there was 4,018 km^2^ of forest, of which 79% was relatively intact with only 15% degraded and 6% severely degraded. In Malaysian Borneo, only a small area of relatively intact forest ecosystems remain, constituting 22% of the total land area. We emphasise that our definition of ‘degradation’ using a 350 m buffer around the roads is conservative. In many areas, and especially on flatter terrain, harvesting extends well beyond this distance (see Supporting Information S1). Consequently, our estimates are likely to underestimate the extent of logged forests.

A previous forest cover map created from 250 m Moderate Resolution Imaging Spectroradiometer (MODIS) data estimated the 2010 forest cover in Malaysian Borneo as 100,150 km^2^ irrespective of condition [34]. Overall, we measured 109,877 km^2^ of forest cover irrespective of condition in 2009, or 108,448 km^2^ adjusted to 2010 assuming a 1.3% annual deforestation rate [34]. While our assessment of overall forest cover was similar to that of a previous satellite mapping study [34], critically we were also able to map forest degradation. We found that of the remaining total forest area in Malaysian Borneo, 62% had been logged once or repeatedly since 1990. Relying solely on moderate resolution mapping to inform policies on forests in this region would result in an enormous 260% overestimate of remaining intact forests. Given that tropical forests lose 20–60% of their standing carbon stocks following selective logging [15]–[19], the monitoring of carbon emissions based on moderate resolution satellite imagery that cannot resolve areas of forest degradation are likely to produce severe underestimates if used in global carbon accounting or REDD schemes.

There has been an increasing focus, especially in the Southeast Asian region, on the importance of protecting logged forests for biodiversity, carbon sequestration and ecosystem health rather than solely concentrating on the conservation of primary forests [35]–[38]. In the Malaysian states of Sabah and Sarawak, there are very few remaining areas of unlogged forest. Indeed, our estimate of 22% is likely to be optimistic because logging in flat terrain usually extends well beyond the 350 m limit we used in this study. Alternatively the forests could have been subject to helicopter logging that could extend the logging front several kilometres from a log pond, although helicopter logging is not widespread. Consequently the actual area of remaining intact forest is probably smaller. Reinforcing the conservative nature of our estimate is the fact that, in Sarawak and Sabah, 8.6% and 5.0% respectively of the area of intact forest is composed of discrete patches less than 0.5 km^2^ in size, while 28% and 19% respectively is less than 5 km^2^ in area. In contrast, in the forests of Brunei, only 2.0% of intact forest is composed of patches less than 0.5 km^2^, and only 5.5% less than 5 km^2^.

Although we did not include Indonesian Borneo in this analysis, moderate resolution forest mapping indicates the situation in the rest of the island of Borneo to be comparable to our findings [34]. Given that such a small area of intact forest ecosystems remains in Malaysian Borneo, it is understandable that ecologists and conservationists in this region are increasingly calling for the protection of logged and degraded forests. While accepting the importance of protecting logged forests, preventing degradation of remaining unlogged forests is also critically important, especially in nations outside South East Asia where substantial areas of unlogged forest remain. The situation in Brunei presents a distinct contrast: 54% of its land area remains covered by relatively intact forest ecosystems – approximately three times that of Sabah and Sarawak. Over the past several decades Brunei has built its wealth from oil and gas extraction, and has largely excluded industrial logging from its borders. This approach has been vastly more successful at forest conservation than has laissez faire logging in Sarawak and Sabah.

Examination of the forests contained in protected area networks in Sarawak and Sabah further supports this point. Only 8% of Sabah’s land area is officially protected unlogged forest. Our findings are even more sobering in Sarawak: protected areas contain 72% intact forest, yet only 3% of the land area is under protected areas. If forest conservation efforts in Sabah and Sarawak relied solely on the protected area networks to prevent loss of primary forest ecosystems, then at most only 8% and 3% of land area in these states would remain intact. It is of concern that in Sarawak, 23% of land the area under protected areas were logged within the study period, whilst in Sabah 30% of protected areas have been logged.

Despite the results reported here, there remains positive news for forest conservation. Although a system of maintaining a small patchwork of ‘protected areas’ largely fails to protect intact forest ecosystems in Malaysian Borneo, neighboring Brunei still retains 54% of land area covered by relatively intact forests. With alternative income sources, such as through voluntary and compliance carbon offset projects [39], Malaysia and other countries might increase their efforts to protect remaining intact forest and to increase carbon stocks on previously logged forests by excluding further logging. For other oil or mineral rich forested nations that have not degraded forest ecosystems to a point where only a small proportion remains, in the long term, the better strategy for preserving forest ecosystems is to keep loggers out altogether rather than to rely on a protected area network or attempts to reform harvesting practices.

After 20 years of attempting to reform harvesting practices in tropical logging operations, the ITTO now acknowledges that more than 90% of logging of natural tropical forests that are intended to remain permanently in the forest estate is unsustainable [40]. According to the ITTO, sustainability of logging of natural forests was defined with respect to maintaining wood production, future forest productivity, and preventing undesirable environmental and social outcomes [40]. The history of forestry in Sarawak and Sabah indicates that attempting to reform the logging industry does not result in meaningful forest conservation. A far better approach, as shown in Brunei, is to prevent logging of natural forests in the first instance, or in places where logging has occurred, to exclude further logging from what remains. In Sarawak and Sabah in particular, and globally across the tropics [3], the crisis in tropical forests is now so severe that any further sacrifice of primary ecosystems to the industrial logging industry ought to be out of the question for all who seriously seek to maintain biodiversity and forest ecosystems.

## Supporting Information

Figure S1 **Protected areas in Sarawak and Sabah, obtained from the World Database on Protected Areas.**
(TIF)

Figure S2 **Map of ‘tree/palm’ cover in 2009 obtained from the CLASlite, for Sabah, Sarawak and Brunei.**
(TIF)

Figure S3 **Forest cover in 2009 with mangroves, plantations and regrowth on previously cleared land shown separately, and major cloud gaps filled.**
(TIF)

Table S1 **Date recorded, and path and row for each Landsat scene used to generate the base tree/palm cover for Sabah and Sarawak.**
(DOCX)

Table S2 **Date of capture, and path and row for each Landsat scene used to digitise plantations and cleared areas, in addition to roads in Sabah and Sarawak.**
(DOCX)

Supporting Information S1 **Examples of the peat swamp drainage and logging, and examples of the conservative 350 m road buffer.**
(DOCX)
