# Supplementary figures and images for: Extreme Differences in Forest Degradation in Borneo: Comparing Practices in Sarawak, Sabah, and Brunei
Source: PLoS One. 2013 Jul 17;8(7):e69679. doi: 10.1371/journal.pone.0069679 (PMC3714267; doi:10.1371/journal.pone.0069679)

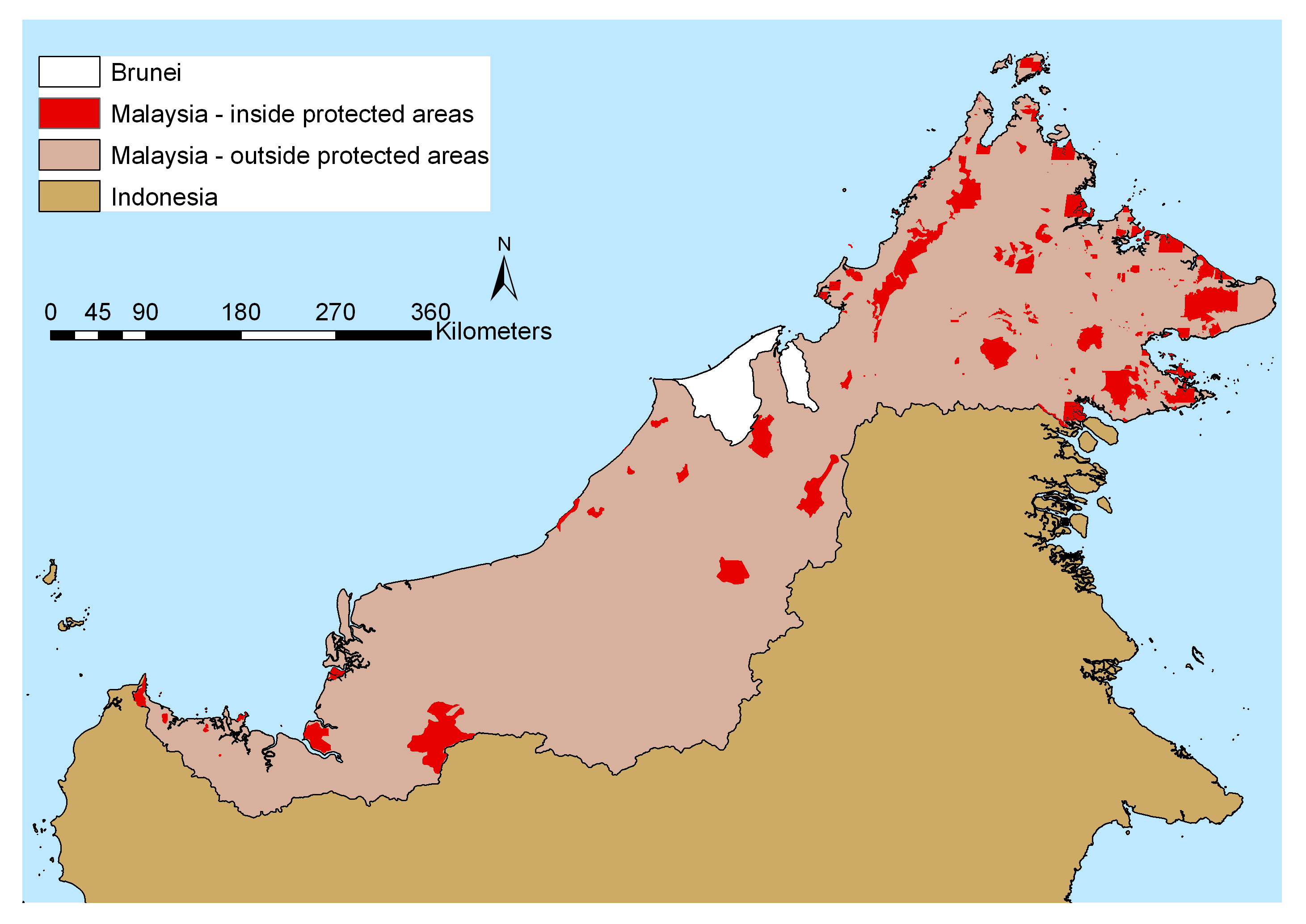

Supplement: Figure S1 — Protected areas in Sarawak and Sabah, obtained from the World Database on Protected Areas. (TIF) [file pone.0069679.s001.tif]

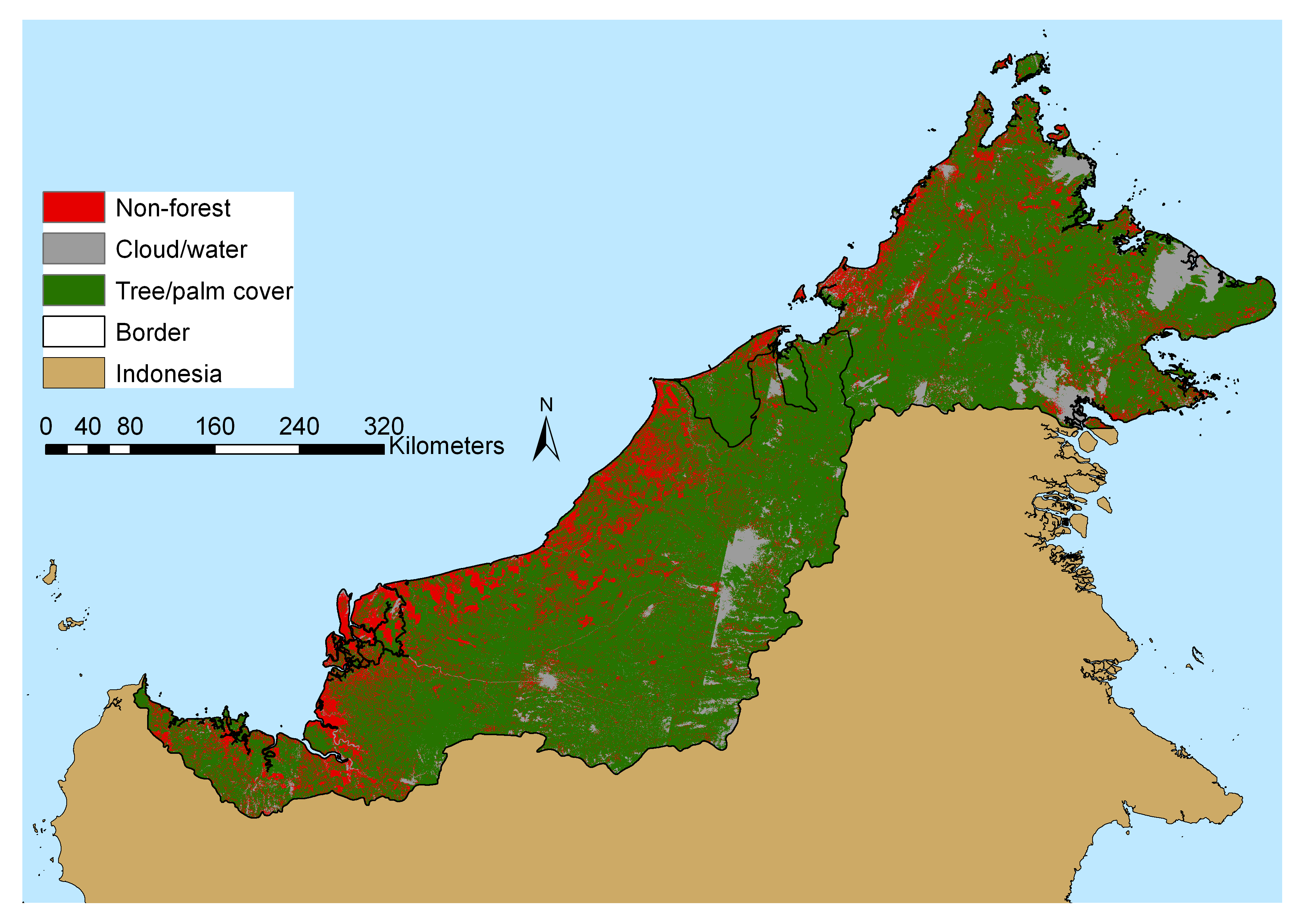

Supplement: Figure S2 — Map of ‘tree/palm’ cover in 2009 obtained from the CLASlite, for Sabah, Sarawak and Brunei. (TIF) [file pone.0069679.s002.tif]

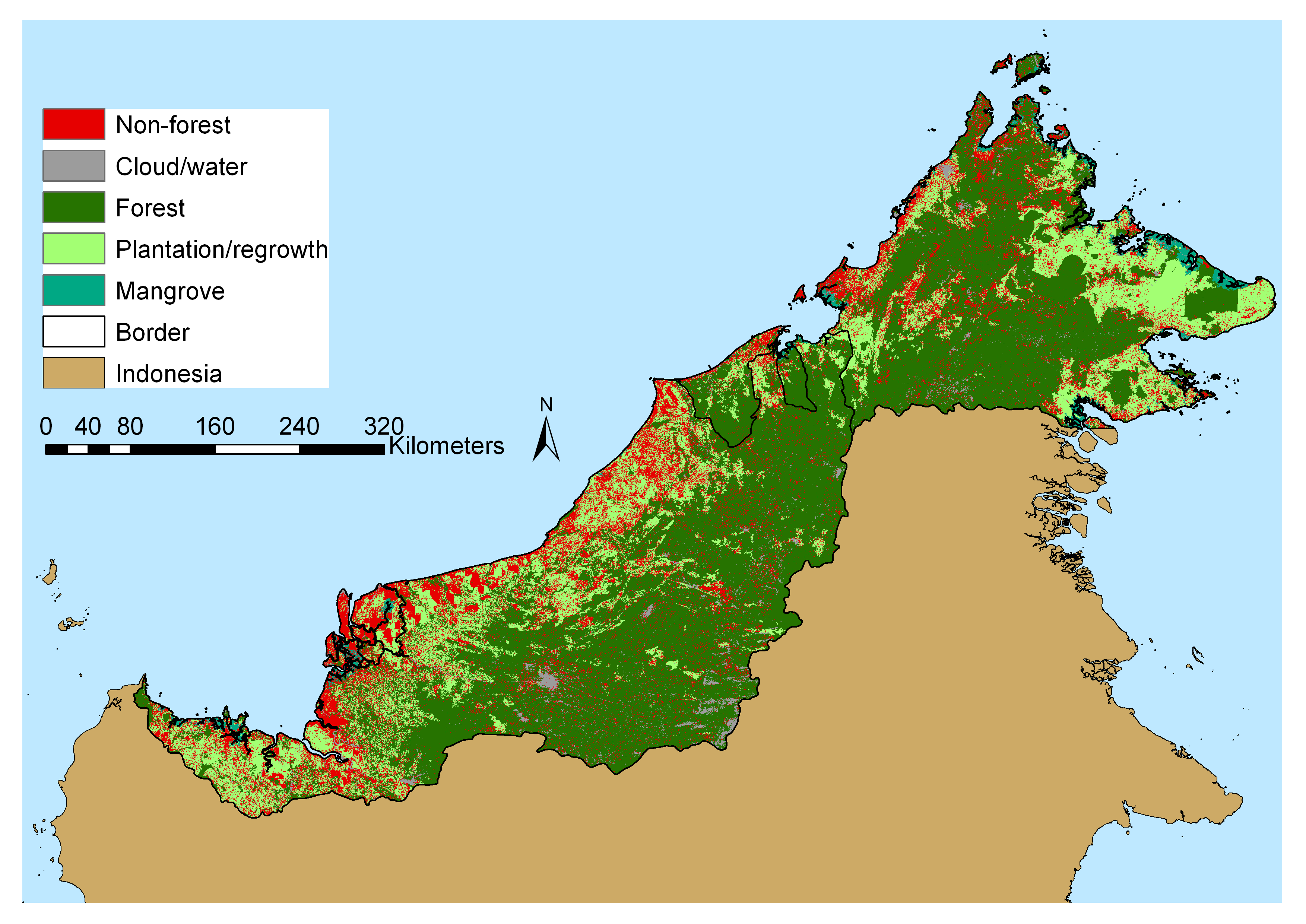

Supplement: Figure S3 — Forest cover in 2009 with mangroves, plantations and regrowth on previously cleared land shown separately, and major cloud gaps filled. (TIF) [file pone.0069679.s003.tif]
