# Supplementary material for: Extreme Differences in Forest Degradation in Borneo: Comparing Practices in Sarawak, Sabah, and Brunei
Source: PLoS One. 2013 Jul 17;8(7):e69679. doi: 10.1371/journal.pone.0069679 (PMC3714267; doi:10.1371/journal.pone.0069679)
Supplement: Table S1 — Date recorded, and path and row for each Landsat scene used to generate the base tree/palm cover for Sabah and Sarawak. (DOCX) [file pone.0069679.s004.docx]

**Table S1.** Date recorded, and path and row for each Landsat scene used to generate the base tree/palm cover for Sabah and Sarawak.

| **Path** | **Row** | **Day** | **Month** | **Year** |
| --- | --- | --- | --- | --- |
| 116 | 56 | 4 | 8 | 2009 |
| 116 | 57 | 4 | 8 | 2009 |
| 116 | 57 | 29 | 3 | 2009 |
| 117 | 55 | 11 | 10 | 2008 |
| 117 | 56 | 11 | 8 | 2009 |
| 117 | 57 | 10 | 2 | 2005 |
| 118 | 55 | 6 | 11 | 2009 |
| 118 | 56 | 28 | 4 | 2009 |
| 118 | 57 | 3 | 9 | 2009 |
| 118 | 58 | 26 | 6 | 2007 |
| 118 | 59 | 10 | 2 | 2010 |
| 119 | 57 | 6 | 11 | 2005 |
| 119 | 57 | 26 | 11 | 2008 |
| 119 | 58 | 10 | 7 | 2001 |
| 119 | 58 | 3 | 7 | 2007 |
| 119 | 59 | 3 | 7 | 2007 |
| 120 | 58 | 31 | 7 | 2009 |
| 120 | 59 | 31 | 7 | 2009 |
| 121 | 59 | 22 | 6 | 2004 |
