# Supplementary material for: Extreme Differences in Forest Degradation in Borneo: Comparing Practices in Sarawak, Sabah, and Brunei
Source: PLoS One. 2013 Jul 17;8(7):e69679. doi: 10.1371/journal.pone.0069679 (PMC3714267; doi:10.1371/journal.pone.0069679)
Supplement: Table S2 — Date of capture, and path and row for each Landsat scene used to digitise plantations and cleared areas, in addition to roads in Sabah and Sarawak. (DOCX) [file pone.0069679.s005.docx]

Table S2. Date of capture, and path and row for each Landsat scene used to digitise plantations and cleared areas, in addition to roads in Sabah and Sarawak.

| **Path** | **Row** | **Day** | **Month** | **Year** |
| --- | --- | --- | --- | --- |
| 116 | 56 | 12 | 9 | 2000 |
| 116 | 56 | 18 | 12 | 2009 |
| 116 | 57 | 6 | 5 | 1990 |
| 116 | 57 | 7 | 11 | 2000 |
| 116 | 57 | 5 | 3 | 2006 |
| 117 | 55 | 6 | 5 | 2000 |
| 117 | 55 | 19 | 8 | 2009 |
| 117 | 56 | 22 | 5 | 1991 |
| 117 | 56 | 3 | 8 | 2006 |
| 117 | 56 | 19 | 8 | 2009 |
| 117 | 57 | 26 | 6 | 2001 |
| 117 | 57 | 17 | 9 | 2005 |
| 117 | 57 | 3 | 8 | 2009 |
| 118 | 56 | 14 | 6 | 1991 |
| 118 | 56 | 19 | 5 | 2002 |
| 118 | 56 | 24 | 9 | 2008 |
| 118 | 56 | 10 | 8 | 2009 |
| 118 | 57 | 25 | 2 | 2001 |
| 118 | 57 | 19 | 5 | 2002 |
| 118 | 57 | 22 | 5 | 2009 |
| 118 | 58 | 25 | 2 | 2001 |
| 118 | 58 | 16 | 5 | 2004 |
| 118 | 58 | 3 | 3 | 2009 |
| 118 | 58 | 22 | 5 | 2009 |
| 118 | 59 | 28 | 12 | 1990 |
| 118 | 59 | 16 | 7 | 2000 |
| 118 | 59 | 2 | 9 | 2000 |
| 118 | 59 | 3 | 3 | 2009 |
| 118 | 59 | 19 | 3 | 2009 |
| 118 | 60 | 28 | 12 | 1990 |
| 118 | 60 | 19 | 5 | 2008 |
| 118 | 61 | 2 | 9 | 2000 |
| 118 | 61 | 22 | 5 | 2009 |
| 118 | 62 | 16 | 7 | 2000 |
| 118 | 62 | 20 | 8 | 2001 |
| 118 | 62 | 7 | 8 | 2005 |
| 118 | 62 | 7 | 6 | 2006 |
| 118 | 62 | 5 | 8 | 2007 |
| 118 | 62 | 19 | 5 | 2008 |
| 119 | 57 | 18 | 4 | 1991 |
| 119 | 57 | 10 | 7 | 2001 |
| 119 | 57 | 11 | 6 | 2005 |
| 119 | 57 | 9 | 2 | 2010 |
| 119 | 58 | 10 | 7 | 2001 |
| 119 | 58 | 14 | 8 | 2005 |
| 119 | 58 | 1 | 8 | 2009 |
| 119 | 58 | 9 | 2 | 2010 |
| 119 | 59 | 10 | 7 | 2001 |
| 119 | 59 | 14 | 8 | 2005 |
| 119 | 61 | 15 | 1 | 2001 |
| 119 | 61 | 11 | 4 | 2003 |
| 119 | 61 | 15 | 3 | 2005 |
| 120 | 58 | 25 | 4 | 1991 |
| 120 | 58 | 31 | 8 | 2000 |
| 120 | 58 | 2 | 6 | 2002 |
| 120 | 58 | 28 | 11 | 2009 |
| 120 | 59 | 25 | 4 | 1991 |
| 120 | 59 | 31 | 8 | 2000 |
| 120 | 59 | 21 | 8 | 2002 |
| 120 | 59 | 1 | 7 | 2004 |
| 120 | 59 | 16 | 8 | 2006 |
| 120 | 59 | 8 | 8 | 2009 |
| 120 | 59 | 20 | 5 | 2009 |
| 121 | 59 | 24 | 6 | 1990 |
| 121 | 59 | 18 | 5 | 2000 |
| 121 | 59 | 22 | 6 | 2004 |
| 121 | 59 | 14 | 7 | 2006 |
| 121 | 59 | 23 | 6 | 2007 |
| 121 | 59 | 8 | 5 | 2008 |
| 121 | 59 | 30 | 7 | 2009 |
| 116 | 56 | 19 | 7 | 2009 |
| 116 | 56 | 4 | 8 | 2009 |
| 116 | 57 | 29 | 3 | 2009 |
| 116 | 57 | 4 | 8 | 2009 |
| 117 | 55 | 11 | 10 | 2008 |
| 117 | 56 | 11 | 8 | 2009 |
| 117 | 57 | 20 | 2 | 2005 |
| 118 | 55 |  |  | 2009 |
| 118 | 56 | 28 | 4 | 2009 |
| 118 | 57 | 15 | 6 | 2009 |
| 118 | 57 | 3 | 9 | 2009 |
| 118 | 58 | 26 | 6 | 2007 |
| 118 | 59 | 10 | 2 | 2010 |
| 119 | 57 | 26 | 11 | 2008 |
| 119 | 58 | 3 | 7 | 2007 |
| 119 | 59 | 3 | 7 | 2007 |
| 120 | 58 | 31 | 7 | 2009 |
| 120 | 59 | 31 | 7 | 2009 |
