# Supplementary material for: Extreme Differences in Forest Degradation in Borneo: Comparing Practices in Sarawak, Sabah, and Brunei
Source: PLoS One. 2013 Jul 17;8(7):e69679. doi: 10.1371/journal.pone.0069679 (PMC3714267; doi:10.1371/journal.pone.0069679)
Supplement: Supporting Information S1 — Examples of the peat swamp drainage and logging, and examples of the conservative 350 m road buffer. (DOCX) [file pone.0069679.s006.docx]

## Peat swamp logging and conversion

In Sarawak especially, a system of intensive drainage and harvesting of peat swamp forests occurs usually prior to conversion for oil palm. Conversion of peat swamp forests involves building a regular criss-cross network of ‘railway’ channels for log removals, usually followed by drainage and clearance for oil palm. Logs are harvested from swamp forests and transported down channels prior to drainage and replacement with oil palm. Examples of the peat swamp forest drainage and conversion process as it appears in Landsat imagery is shown in Figure S4, and on-ground photographs of the ‘railway’ channels and system of log removal are shown in Figures S5 and S6.

**
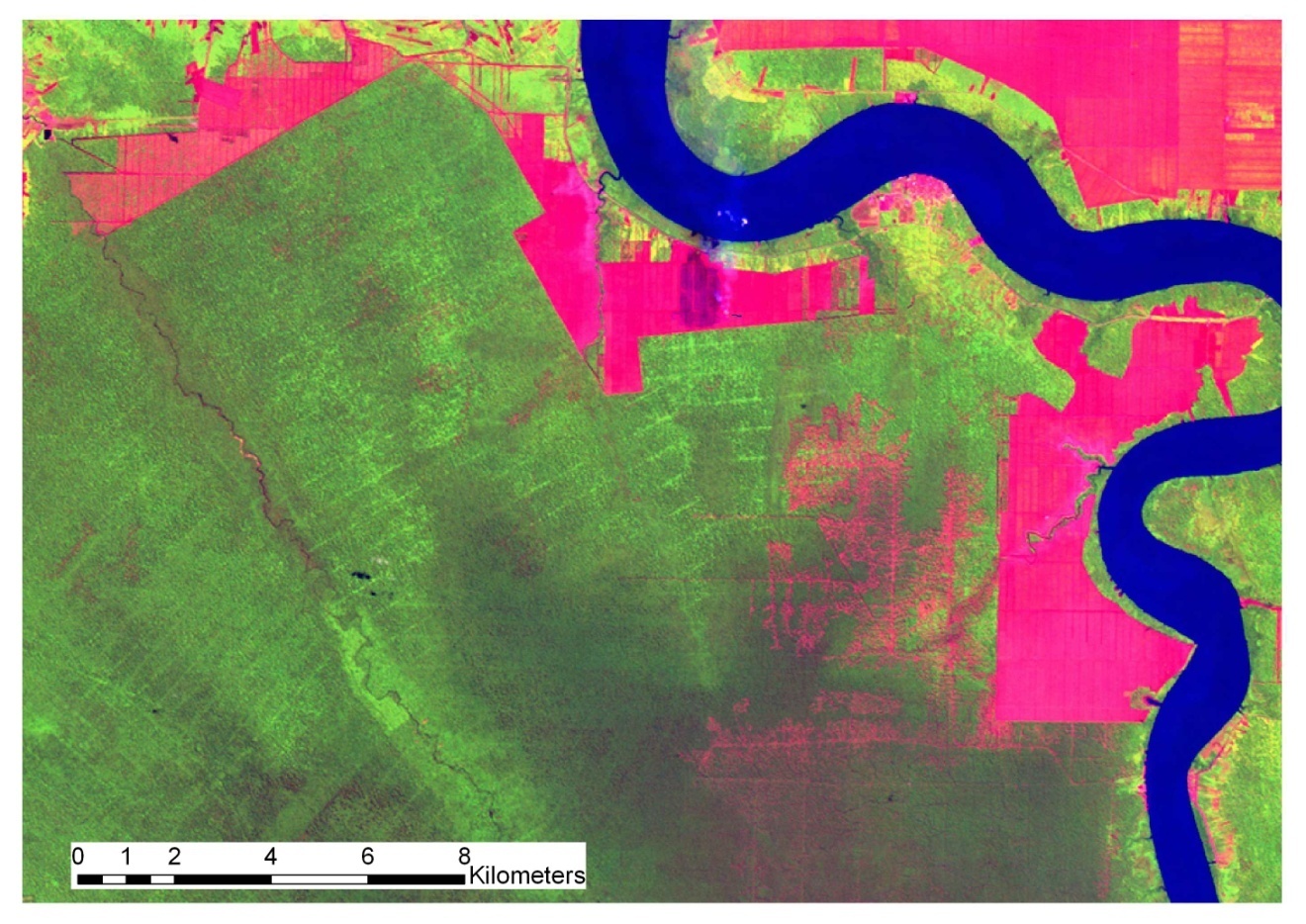
**

**Figure S4. Infra-red enhanced image showing oil palm and peat swamp forest conversion in Sarawak. Bare ground and recently cleared land appears as red/pink. Oil palm plantations appear as light green/red with regular rectangle shaped roading patterns. The criss-cross pattern of roading elsewhere represents the ‘railways’ of roads and drainage channels through peat swamps.**


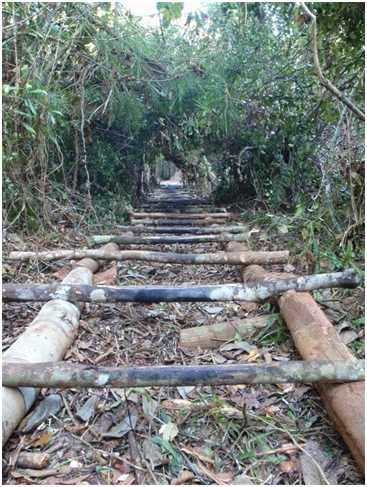


**Figure S5. Example of a ‘railway’ channel built through peat swamp forests for log transportation.**


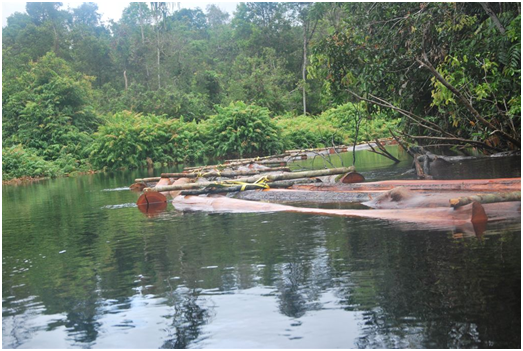


**Figure S6. Example of harvested logs removed from peat swamp forest via a river.**

## Conservative 350 m road buffer

**
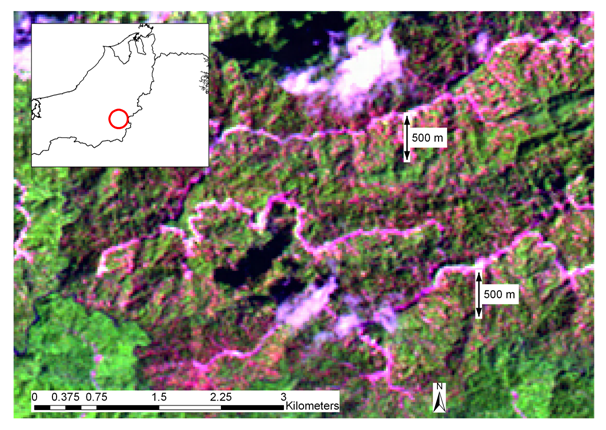
**

**Figure S7. Infra-red colour enhanced Landsat image of recent harvesting in hilly terrain in Sarawak. Cleared areas including roads, skid tracks and canopy gaps are shown in pink. Harvesting in the form of skid tracks and canopy gaps can be seen extending further than 350 m.**


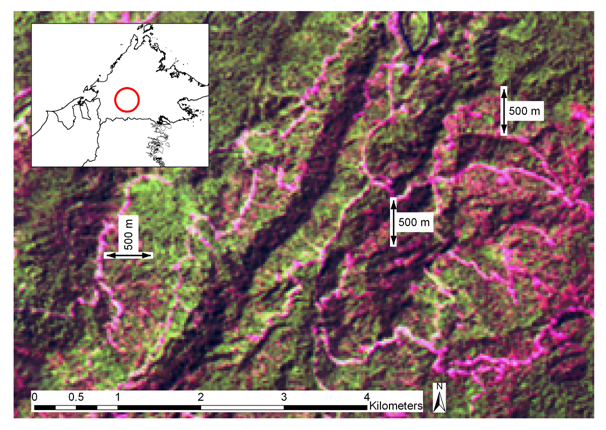


**Figure S8. Infra-red colour enhanced Landsat image of recent harvesting in hilly terrain in Sabah. Cleared areas including roads, skid tracks and canopy gaps are shown in pink. Harvesting in the form of skid tracks and canopy gaps can be seen extending further than 350 m.**


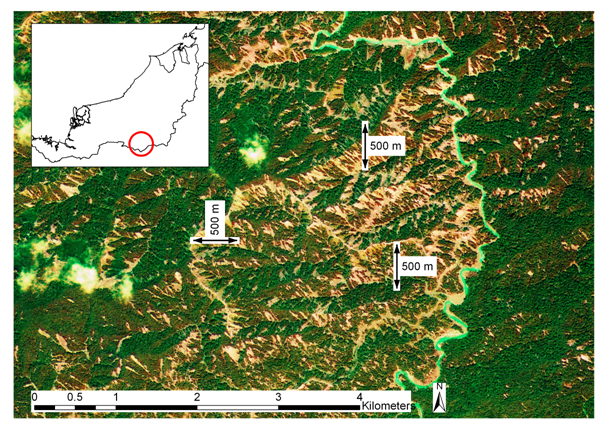


**Figure S9. True colour Satellite Pour l’Observation de la Terre (SPOT) image showing recent harvesting in hilly terrain in Sarawak, near the border with Indonesia. Cleared areas including roads, skid tracks and canopy gaps are shown in brown. Harvesting in the form of skid tracks and canopy gaps can be seen extending further than 350 m.**
